# The Effect of Bariatric Surgery on the Spectrum of Fatty Liver Disease

**DOI:** 10.1155/2016/2059245

**Published:** 2016-09-29

**Authors:** Jordan J. Nostedt, Noah J. Switzer, Richdeep S. Gill, Jerry Dang, Daniel W. Birch, Christopher de Gara, Robert J. Bailey, Shahzeer Karmali

**Affiliations:** ^1^Department of Surgery, University of Alberta, Edmonton, AB, Canada; ^2^Department of Surgery, University of Calgary, Calgary, AB, Canada; ^3^Centre for the Advancement of Minimally Invasive Surgery (CAMIS), Royal Alexandra Hospital, Edmonton, AB, Canada; ^4^Division of Gastroenterology, University of Alberta, Edmonton, AB, Canada

## Abstract

Nonalcoholic fatty liver disease is becoming one of the most common causes of liver disease in the western world. The most significant risk factors are obesity and the metabolic syndrome for which bariatric surgery has been shown to be an effective treatment. However, the effects of bariatric surgery on nonalcoholic fatty liver disease, specifically liver fibrosis and cirrhosis, are not well established. We review published bariatric surgery outcomes with respect to nonalcoholic liver disease. On the basis of this review we suggest that bariatric surgery may provide a viable treatment option for the treatment of nonalcoholic fatty liver disease, including patients with fibrosis and compensated cirrhosis, and that this topic should be a target of future investigation.

## 1. Introduction

Nonalcoholic fatty liver disease (NAFLD) is becoming the most common cause of liver disease in the US affecting one-third of the general population and greater than 75% of obese patients [1]. NAFLD is a continuum of disease ranging from hepatic steatosis alone to nonalcoholic steatohepatitis (NASH) and the most severe end of the spectrum involves fibrosis and cirrhosis [2]. It has been suggested that liver fibrosis is likely to manifest in up to 1/3 of NAFLD patients within 4 years without treatment [3]. Obesity and the metabolic syndrome are the primary risk factors for NAFLD. While current guidelines in the management of obesity recommend diet and exercise as first line treatment [2], however it is now well known that weight loss achieved through lifestyle modification tends to be transient with most patients relapsing [1]. Bariatric surgery has proven to be an effective, sustained treatment of severe obesity. Here, we review the effects of bariatric surgery on the spectrum of NAFLD, from steatosis to cirrhosis, and discuss whether bariatric surgery should be utilized as a primary indication for nonalcoholic fatty liver disease.

## 2. Methods

A search of all electronic databases was performed. English speaking articles and conference abstracts containing the search terms “fatty liver OR non-alcoholic fatty liver disease OR steatosis OR non-alcoholic steatohepatitis” AND “bariatric surgery OR gastric bypass OR gastric band^*∗*^ OR sleeve gastrectomy OR bariatric” were analyzed for inclusion into this mini review.

## 3. Discussion

The prevalence of NAFLD ranges from 17% to 51% in North America depending on the population studied and the method of measurement [2]. Specifically, severely obese patients undergoing bariatric surgery demonstrate NAFLD rates as high as 90% [2]. Studies that have evaluated the effect of surgery on NAFLD have been quite heterogeneous, with respect to procedure performed, follow-up, and reporting of histological changes [1].

### 3.1. Nonalcoholic Fatty Liver Disease: Steatosis

Multiple studies have demonstrated improvement in steatosis following bariatric surgery. Caiazzo et al. showed a statistically significant reduction in steatosis following bariatric surgery based on liver biopsy samples [4]. In another prospective trial, liver biopsy demonstrated improvement of steatosis in all 26 patients, including 12 of the 26 exhibiting improvement of greater than 2 histopathological stages [5]. These results have been consistent in the literature. A 2015 systematic review by Bower et al. showed a 50% mean reduction in the incidence of steatosis across 16 trials that measured pre- and postoperative steatosis [6]. In addition to this meta-analysis, Taitano et al. study, also published in 2015, demonstrates significant improvement in steatosis following bariatric surgery [7].

### 3.2. Nonalcoholic Steatohepatitis (NASH)

Studies have shown that NASH patients undergoing bariatric surgery have significant improvement in both steatosis and hepatocyte ballooning along with resolution of NASH reported at 1 and 5 years postoperatively [8]. In a study by Dixon et al. [9] complete resolution of NASH was seen in 82% of patients undergoing bariatric surgery. In addition to histological improvements, bariatric surgery was also shown to improve liver functional capacity [10].

However, despite these observed benefits, a Cochrane review done in 2010 reported insufficient high quality evidence to definitively assess both positive and negative results of bariatric surgery as a treatment option for patients with NASH [11]. This led to recent guidelines for the management of NAFLD published in 2012, which state that “it is premature to consider foregut bariatric surgery as an established option to treat NASH” [2]. A recent meta-analysis performed in 2015 still supports improvements in liver histology and reductions in liver enzymes after surgery; however it too calls for higher-level evidence prior to making powerful conclusions on the benefits of bariatric surgery for NAFLD [6]. Therefore, there remains paucity in guidelines established for recommendation of bariatric surgery for the specific indication of NAFLD/NASH.

### 3.3. Fibrosis

The literature is mixed with respect to the effect of surgery on fibrosis specifically. Some studies suggest a possible increase in hepatic fibrosis in patients undergoing bariatric surgery [2], thought to be secondary to the increase in pro-inflammatory cytokines at the time of surgery [12]. However, there is now evidence demonstrating no significant worsening of fibrosis in patients with NASH postoperatively. Relative to baseline fibrosis scores, 80% of patient's fibrosis stage was unchanged or regressed 5 years postoperatively as assessed by liver biopsy and semiquantitative fibrosis scoring by a blinded pathologist [8]. Mathurin et al. [8] showed that 95% of patients with fibrosis at the time of surgery kept a fibrosis score no higher than 1 suggesting little disease progression postoperatively. Klein et al. in a study of extremely obese patients (BMI > 50) undergoing gastric bypass found that although fibrosis did not change and the hepatic gene expression of profibrogenic cytokines was reduced after surgery suggesting a benefit to reducing fibrogenesis [13]. Additionally, Vargas et al. found that fibrosis scores either improved or remained stable with no worsening of fibrosis seen in paired liver biopsies following bariatric surgery [5]. More recently, Taitano et al. found fibrosis to resolve or improve in 60% of patients [7].

Adipose tissue has now been shown to be a source of proinflammatory cytokines [14]. The majority of weight loss after bariatric surgery is adipose tissue leading to decreased production of these factors and thus also reducing fibrosis. The reduction in adipocytes also improves insulin resistance [15] which is also an important mechanism due to the association of insulin resistance with oxidative stress and development of fibrosis [16]. It appears then that bariatric surgery can be safely performed in patients with fibrosis, but more data is needed prior to recommending it as a potential management tool in the disease course.

### 3.4. Compensated Cirrhosis

Cirrhosis is incidentally discovered in only 1–3% of bariatric surgery patients [1] and the data on cirrhotics is still fairly limited [17]. However this is a topic of increasing interest as it has now been suggested by some that in patients with compensated cirrhosis, bariatric surgery is well tolerated and may actually be beneficial [1]. This was recently reviewed by Jan et al. [18]. A total of eleven studies of bariatric surgery for patients with cirrhosis were reviewed showing a general trend toward higher risk of complication and mortality in cirrhotic patients. However, amongst the studies included in this review, several reported mortality rates of zero and minimal adverse complications [19, 20] leading to the authors conclusion that “evidence is insufficient to make any clear recommendations” [18]. It should also be highlighted that, in the study by Mosko and Nguyen, mortality rates were reduced when analysis was limited to compensated liver failure and high volume centers [21]. Taken together, we are unable to make a recommendation on performing bariatric surgery in a patient with compensated liver failure.

### 3.5. Decompensated Cirrhosis

There are limited published studies on decompensated cirrhotics undergoing bariatric surgery. However, we know from the general surgical literature that cirrhosis presents many operative challenges including extensive hemorrhage, coagulopathy, reduced hepatic blood flow leading to liver dysfunction, and decreased metabolism of drugs [22]. Bariatric patients often have multiple comorbidities including type 2 diabetes, hypertension, dyslipidemia, and obstructive sleep apnea and although bariatric surgery has been proven to have excellent outcomes for these comorbidities [23], these conditions when combined with decompensated cirrhosis place these individuals at too high of surgical risk to recommend the procedure. Mortality in this population showed an odds ratio of 21.2 relative to noncirrhotic patients [21].

## 4. Conclusion

The expanding positive results in bariatric surgery with respect to the spectrum of NAFLD, including liver fibrosis and compensated cirrhosis, hint that it may be time to start utilizing bariatric surgery for treatment of these conditions. At present time there is not sufficient high quality evidence to recommend bariatric surgery for the treatment of NAFLD, hepatic fibrosis, or compensated cirrhosis; however, these should be the target of future investigation. Despite limited published data, the obese patients with decompensated cirrhosis remain to be poor candidates for bariatric surgery due to significant mortality rates.
